# Dr. Joseph W. Howe

**Published:** 1890-07

**Authors:** 


Dr. Joseph W. Howe, of New York, died at sea, of apoplexy, June 7, 1890, on board the Cunard steamer Umbria, which arrived at Queenstown June 14th. His body has been preserved and will be returned to New York for burial. The *Boston Medical and Surgical Journal* publishes the following:

Dr. Howe was born at New Brunswick, September 30, 1843, and when a young man became a reporter for the *Colonial Times*, of which his father was editor and proprietor. He subsequently studied medicine, and in 1866 was graduated from the medical department of the University of the City of New York. After graduation he served as interne at Bellevue Hospital, on the surgical side, and in 1868 was appointed Clinical Professor of Surgery in the University Medical School, a position which he held until 1882. In the meanwhile he became consulting surgeon to the Charity Hospital, Blackwell's Island, and visiting surgeon to St. Francis Hospital, positions which he retained subsequently. Among the works which he wrote were Winter Homes for Invalids, and Emergencies and How to Treat Them. He was a delegate to the International Medical Congress at Copenhagen in 1884, and was on his way to the Tenth International Congress at Berlin at the time of his death. Dr. Howe ranked high as a surgeon, and was personally very popular. He leaves a widow and one daughter, and his untimely taking off will be deeply regretted by the profession and a large circle of friends.

